# “Cyclist at 12 o’clock!”: a systematic review of in-vehicle advanced driver assistance systems (ADAS) for preventing car-rider crashes

**DOI:** 10.3389/fpubh.2024.1335209

**Published:** 2024-02-19

**Authors:** Sergio A. Useche, Mireia Faus, Francisco Alonso

**Affiliations:** Research Institute on Traffic and Road Safety (INTRAS), University of Valencia, Valencia, Spain

**Keywords:** ADAS, inter-user crashes, vehicles, bicycles, injury, riding safety

## Abstract

**Introduction:**

While Advanced Driver Assistance Systems (ADAS) have become a prominent topic in road safety research, there has been relatively little discussion about their effectiveness in preventing car collisions involving specific vulnerable road users, such as cyclists. Therefore, the primary objective of this systematic literature review is to analyze the available evidence regarding the effectiveness of in-vehicle ADAS in preventing vehicle collisions with cyclists.

**Methods:**

To achieve this goal, this systematic review analyzed a selection of original research papers that examined the effectiveness of ADAS systems in preventing car-cyclist collisions. The review followed the PRISMA protocol, which led to the extraction of 21 eligible studies from an initial pool of 289 sources indexed in the primary scientific literature databases. Additionally, word community-based content analyses were used to examine the research topics and their links within the current scientific literature on the matter.

**Results:**

Although the current number of studies available is still scarce (most sources focus on car-motorcyclist or car-pedestrian crashes), the overall quality of the available studies has been reasonably good, as determined by the selected evaluation methods. In terms of studies’ outcomes, the literature supports the value of in-vehicle ADAS for preventing car-cyclist crashes. However, threatful side effects such as unrealistic expectations of these systems and users’ overconfidence or desensitization are also highlighted, as well as the need to increase driver training and road user awareness.

**Conclusion:**

The results of this study suggest that Advanced Driver Assistance Systems have significant potential to contribute to the prevention of driving crashes involving cyclists. However, the literature emphasizes the importance of concurrently enhancing user-related skills in both ADAS use and road-user interaction through educational and training initiatives. Future research should also address emerging issues, such as ADAS-related behavioral ergonomics, and conduct long-term effectiveness assessments of ADAS in preventing car-cycling crashes and their subsequent injuries.

**Systematic review registration:**

PROSPERO, unique identifier CRD42024505492, https://www.crd.york.ac.uk/prospero/display_record.php?RecordID=505492.

## 1 Introduction

Despite the several ongoing efforts to reduce traffic crashes and injuries involving cyclists, the latest increases in their number -transcending borders and affecting all regions- have put in evidence the need to strengthen their prevention (1). Globally, an estimated 69,000 people are killed each year while cycling and another 11 million cyclists are injured in this type of crash (2, 3). Thus, pedestrians and cyclists account for 26% of all road traffic fatalities, figures that increase to 44% in Africa and up to 36% in the Eastern Mediterranean (4). Specifically, in countries such as the United States, fatality rates per kilometer increased in recent years by 33% for cyclists, although they remained stable in other regions such as Germany, the United Kingdom or Denmark (5).

Among many latent risks for cyclists, road conflicts with motorized users and other threatening common situations put in manifest their several shortcomings in terms of riders’ passive safety (i.e., related to actual post-crash consequences), thus explaining a considerable proneness to suffer severe injuries as a consequence of rising crashes among them (6). This is usually reflected in fatality data in several regions. As a figure, in countries such as Spain, about four out of every 10 cyclists (namely 42%) dead in traffic incidents were killed on conventional single-carriageway roads, where motor vehicles and bicycles necessarily share space (7).

Also, and speaking in task-related terms, cyclists represent a particularly vulnerable group on the roads due to a number of intrinsic and extrinsic factors. In this regard, in addition to the special risk in overtaking situations, their direct exposure to the environment and the lack of protective bodywork makes them more vulnerable targets in case of collisions (8, 9). The lack of turn signals and brakes on bicycles, compared to motor vehicles, can make it difficult for drivers to anticipate their movements (10). In addition, road conditions, lack of safe infrastructure for cyclists (11, 12), and lack of respect by some drivers toward this road group can significantly increase their risk of being involved in traffic crashes (13, 14).

As for road user types, in other road contexts or situations, conventional cars represent the means of transport mostly involved in collisions with cyclists. For instance, official figures indicate that, in European countries such as France, 66% of deaths in this road group occur in passenger car accidents (15). In Germany, meanwhile, 75% of on-road cyclist fatalities are linked to problematic interaction with cars (16). In this regard, mixed traffic involving motor vehicles and vulnerable road users poses a high risk as cyclists and pedestrians can be commonly (and seriously) injured or killed at speeds of 40 km/h or higher, speeds that are reached in many countries in urban areas (17, 18). Nevertheless, during the last few years ADAS designed specifically to prevent impacts with cyclists have been introduced into new vehicles, the effect of which is anticipated to provide both crash prevention and injury mitigation benefits to bicycle riders.

### 1.1 Are ADAS relevant contributors for current and future road safety?

Two of the currently ‘hottest’ topics in crash prevention are both the ‘if’ and ‘how’ of how Advanced Driver Assistance Systems (ADAS) may contribute to reducing road fatalities among vulnerable road users. And the gaps are evident when comparing their impact between car driving and cycling: although ADAS are now relatively common among four-wheeled vehicles, the number of bicycles incorporating them remains scarce in the market, even in high-income economies (19). Similarly, the more common ADAS-related safety studies are those conducted among car drivers. Still, their influence on road safety figures remains relevant as, all in all, the existing literature highlights how vehicle technology holds great potential to improve road safety globally, and the core reason explaining it could be that they contribute to preventing and counteracting human failures, possibly implicated in up to 90% of crashes (20).

Furthermore, safety-related literature highlights that, although crash-prevention technology is still limited, it might help strengthen road users’ training, prevent road conflicts among different groups of them, and increase the effectiveness of road safety measures to protect vulnerable road users, including cyclists and pedestrians (19, 21). In this regard, the implementation of Advanced Driver Assistance Systems (ADAS) devices, designed to alert drivers to the presence of cyclists in their surroundings, emerges as a potentially effective and universal solution to address this global challenge (21). These devices can leverage various technologies, including cameras, radar, and proximity sensors, to detect the presence of cyclists and provide the driver with an early warning. Upon receiving a notification about the presence of a cyclist, the driver has the opportunity to take preventive measures, such as slowing down, providing more space to the rider, or waiting until it is safe to overtake (22).

Research conducted in various regions highlights the impact of ADAS systems on reducing fatalities involving vulnerable users. For instance, it is assumed that ADAS-related vehicle improvements have been responsible for a 23% reduction in car-pedestrian collisions in Sweden (23). In the United States, vehicles equipped with automatic braking systems were found to have a 43% lower likelihood of being involved in a rear-end collision toward cyclists and other drivers, and cars equipped with lane-keeping assistants had a 9% lower chance of leaving the road (24). In this same line, Cicchino concludes that, if properly used, lane departure warning systems can reduce the fatal crash rates by 86% (25). In addition, Sander and Lubbe estimate that driver warning systems can reduce intersection accidents by up to 50% (26). Also, Seacrist et al. (27) claim that automatic braking is particularly relevant for reducing collisions, with a potential to minimize incidents by 48%, followed by vehicle-to-vehicle communication (38%) and driver monitoring systems (24%). In this line with these figures, during the last few years, ADAS designed specifically to prevent impacts with cyclists have been growingly introduced into new vehicles, the effect of which is anticipated to provide both crash prevention and injury mitigation benefits to bicycle riders (28).

### 1.2 ADAS systems for the prevention of collisions with bicyclists

According to their function and specific features, Advanced Driver Assistance Systems can play a crucial role in preventing collisions between cars and cyclists. These systems incorporate sophisticated technologies that can detect the presence of cyclists and take action to reduce the risk of collisions (29). Several types of ADAS that can potentially be effective in preventing collisions between cars and cyclists. The most common are described below:

- Forward Collision Warning (FCW): this system uses sensors, such as cameras and radar, to detect objects in the vehicle’s path, including cyclists (30). If the system identifies an imminent risk of collision with a cyclist, it issues a visual and audible warning to the driver to take evasive action or reduce speed. The key to FCW’s effectiveness lies in its ability to perform real-time analysis of the collected information. Advanced algorithms constantly process data on the position, speed, and direction of surrounding objects, ensuring accurate and timely detection of any collusion threat (31). The issuance of visual and audible warnings to the driver in the event of an imminent risk enables rapid decision making, providing the opportunity to take evasive action or reduce speed to avoid collision.
- Emergency braking with pedestrian and cyclist detection: this system is an extension of FCW and, instead of simply warning the driver, can automatically activate the brakes if an imminent collision with a cyclist is foreseen (32). Pedestrian and cyclist detection is provided by advanced sensors that constantly monitor the environment. The importance of this system lies in its ability to autonomously anticipate and respond to potential threats. By extending the functionality of FCW, not only are alerts issued to the driver, but in high-risk scenarios with vulnerable road users involved, the system can take direct action to avoid collision. Advanced sensors play a key role in this capability, continuously analyzing the presence and movement of users in the vehicle’s surrounding environment. In this way, it provides an active and rapid response in critical situations (33).
- Blind Spot Detection (BSD): this system use sensors to detect the presence of vehicles, including bicycles, in the car’s blind spots (34). If the driver indicates a turn or lane change while a cyclist is in the blind spot, the system issues a visual or audible alert to prevent a collision. BSD technology relies on advanced sensors that continuously scan the vehicle’s surroundings, identifying the presence of other road users in areas that might escape the driver’s direct field of vision (35). The integration of this technology not only improves the driver’s situational awareness, but also significantly reduces the risk of collisions in situations where visibility is limited.
- Lane Keeping Assist (LKA): this system helps the driver keep the car in its lane, which can be especially important when overtaking cyclists. If the vehicle comes dangerously close to a cyclist or deviates from its lane, the system can intervene in the steering to correct the trajectory (36). LKA technology uses advanced sensors to monitor the vehicle’s position within the lane. When it detects that the car is getting dangerously close to a cyclist or experiencing an unintended lane drift, the system takes corrective action (37). Steering intervention is subtle but effective, helping to keep the vehicle on track and ensuring safe space around cyclists.
- Adaptive Cruise Control (ACC): this system adjusts vehicle speed to maintain a safe distance from vehicles ahead, which also applies to cyclists (38). If a cyclist is in front of the ACC-equipped vehicle, the system will slow down and maintain a safe distance. The importance of this functionality is especially highlighted in situations where the vehicle speed could be inappropriate or potentially dangerous when approaching cyclists. By considering the presence of users around the vehicle, ACC acts proactively, adjusting the speed automatically to avoid risky situations (39).
- Moving object detection systems: These systems use cameras and radar to detect the speed and trajectory of moving objects, such as cyclists. In critical situations, if the system identifies a risk, it can activate instant safety measures, such as automatic braking or issuing alerts to the driver (40). This proactive approach not only improves safety by preventing potential collisions, but also highlights the technology’s ability to dynamically adapt to changing environments, thus providing an additional layer of protection for cyclists and other moving roadway elements.
- Adaptive lighting systems: This system vehicle headlights to better illuminate the presence of cyclists on the road, especially in poor visibility conditions (41). This improves the cyclist’s visibility and allows the driver to react more effectively.

The combination of these ADAS systems helps prevent collisions between cars and cyclists by providing alerts and taking safety measures in risky situations. However, research on the effectiveness of these driver assistance systems, specifically on the cycling population, is limited.

### 1.3 Objective of the systematic review

The core aim of this systematic review of the scientific literature was to comprehensively analyze the evidence on in-vehicle Advanced Driver Assistance Systems (ADAS) for preventing road collisions with cyclists.

As a potential contribution (or set of them), this review may contribute to serve as a reference to synthesize the scientific evidence and provide an overview of the effectiveness and current topics in the implementation of these systems, as well as to identify possible areas for improvement in the protection of cyclists in the traffic environment. Also, it is noteworthy that no previous study has specifically reviewed the literature on this issue.

## 2 Methods

The systematic review developed in this manuscript followed the recommendations of the Cochrane Review Group (42) and the PRISMA 2020 quality standards and protocols (43). The authors of this article conducted the selection, evaluation, and data extraction of the articles individually. Joint discussions were then held to identify articles for inclusion, with final inclusion/exclusion decisions made by consensus.

### 2.1 Protocol and registration

In order to meet the standards protocol for this systematic review, it was registered in PROSPERO (January 24, 2024, ID: 505492). PROSPERO is an international database that registers systematic reviews in (principally, although not exclusively) health and social care. Apart from enhancing transparency, this help to reduce the risk of duplication of the review and strengthens visibility of the current review among other researchers and/or relevant stakeholders in the field.

#### 2.1.1 Definition and scope

The standard purpose of a literature review procedure is to target publications and scientific evidence that provide a comprehensive overview of a certain pre-defined topic, i.e., advanced driving assistance systems (ADAS) used by motor vehicles to prevent collisions with cyclists. In this sense, research on any type of ADAS system may be included as long as its main function is the protection of bicycle users, such as cyclist detection devices, emergency braking systems, blind spot alerts or steering assistance systems, among others.

### 2.2 Eligibility

The research under consideration for this systematic review pertained to the effectiveness of Advanced Driver Assistance Systems (ADAS) in terms of preventing accidents (i.e., traffic crashes), injuries, and fatalities, as well as their influence on modifying driver behavior. Studies that do not explicitly analyze the effectiveness of ADAS systems concerning the safety of cyclists on the road will be excluded. Similarly, research that generally assesses the impact of these systems on vulnerable groups will not be included unless specific results pertaining to bicycle users are provided. Additionally, articles focusing on technological devices installed on bicycles themselves will not be considered. Therefore, this systematic review specifically concentrates on ADAS systems found solely in motor vehicles, such as cars, motorcycles, and trucks, among others.

In terms of geographical coverage, we adopted an ‘open criteria.’ This approach not only avoids limiting research results based on their origin but emphasizes the importance of source quality. This strategy adds value by helping identify countries and/or regions where more research on the topic is being conducted. This also allows to compile and document key findings from the scientific literature, identify potential limitations inherent to this type of study, and conduct a comprehensive discussion of the results.

#### 2.2.1 Information sources

The review process adhered to the recommendations and requirements outlined in the PRISMA 2020 reports for systematic reviews (44). Initially, a scoping review of the literature was conducted, serving as a crucial phase preceding the comprehensive systematic review. This mapping phase aims to understand the extent and variability of the literature in a specific area, facilitating an assessment of the potential and scope of the research objectives. Additionally, it plays a key role in identifying essential terms for subsequent search strategies. During this phase, studies were identified and defined as ‘goldset’ studies, assisting in the identification of relevant search terms.

Electronic searches of databases, including PubMed, Scopus, and Web of Science, were conducted between September 20, 2023, and January 25, 2024. No exclusion criteria based on the year of publication were applied, encompassing all literature published from database creation to the search date. The choice of these databases was guided by their broad support and recognition as reliable indicators of quality within the scientific community. Other sources such as Medline were excluded due to scope-related issues (as this systematic review was framed within the field of technological aids to safe mobility). Google Scholar was also excluded due to its overrepresentation of gray (non-peer-reviewed) literature (45) and concerns about its scientific precision, as noted in preceding literature (46).

Furthermore, we examined other reference lists of previous similar or field-related scoping and systematic reviews of primary research that might have been relevant. However, no eligible results were not detected by our search algorithms.

### 2.3 Search terms and Boolean operators

Search terms (both indexed [e.g., Medical Subject Headings] and keywords) associated with all concepts were independently derived by each author in consultation with a subject matter expert librarian. The collaborative effort ensured a comprehensive approach to the identification of relevant literature covered by the scope of this systematic review.

The review criteria encompassed studies available in both English and Spanish. Consequently, key terms and Boolean search operators were tailored to accommodate these two languages (see Table 1).

**Table 1:** Search strategy for eligible articles.

Search strategy item	Search strategy
Databases	PubMed, Scopus, and Web of Science
Language filter	English and Spanish (indexing languages)
Boolean search operator and Keywords	The identical Boolean search operator was used across all databases. (ADAS AND systems or sistemas) AND (coche-bicicleta OR car-bicycle) AND (motocicleta-bicicleta OR motorcycle-bicycle) AND (camion-bicicleta OR truck-bicycle) AND (proteccion OR prevención OR protection OR prevention) AND (accidentes OR crashes OR accidents)

The search results were exported to Endnote X8 software and subsequently imported into Covidence, a Cochrane technology platform. To curate data sources, duplicates were removed using a standard function applied to the total number of identified records.

For each title/abstract, the three reviewers independently screened for eligibility, adhering to *a priori* inclusion and exclusion criteria. Following title/abstract screening, the same three reviewers independently applied the inclusion and exclusion criteria to the remaining full-text records. Articles not directly related to the research focus were excluded during this phase. To manage potential discrepancies in the selection process, all authors individually evaluated a specific set of titles and abstracts before engaging in discussions to reach a scientific agreement.

Gray literature, including doctoral dissertations, conference papers, editorials, case reports, protocols, or case series, was not excluded, provided it was related to the research objectives. Another eligibility criterion was that articles were available in their entirety for reading, either as open access or through requests made via the institutional library system utilized by the searching authors (UV Trobes).

### 2.4 Data collection

For this study, we employed the descriptive-analytic method proposed by Arksey and O’Malley (46) to critically appraise articles meeting the inclusion criteria. The three reviewers conducted a full-text review of included studies, extracting key data items, including author(s), year of publication, country of study, objectives, methods and sample, results (main findings), and key limitations. The extracted data were systematically recorded in tables and thoroughly documented.

A comprehensive description of the essential conclusions is provided, emphasizing the main findings of the selected articles. To ensure the reliability of our results and mitigate potential bias, studies underwent a quality assessment using the Critical Appraisal Skills Program (CASP). CASP provides a structured framework for critically evaluating the validity of research, aiding in the determination of the overall reliability of the studies included in the review.

## 3 Results

### 3.1 Search results

**Figure 1:**
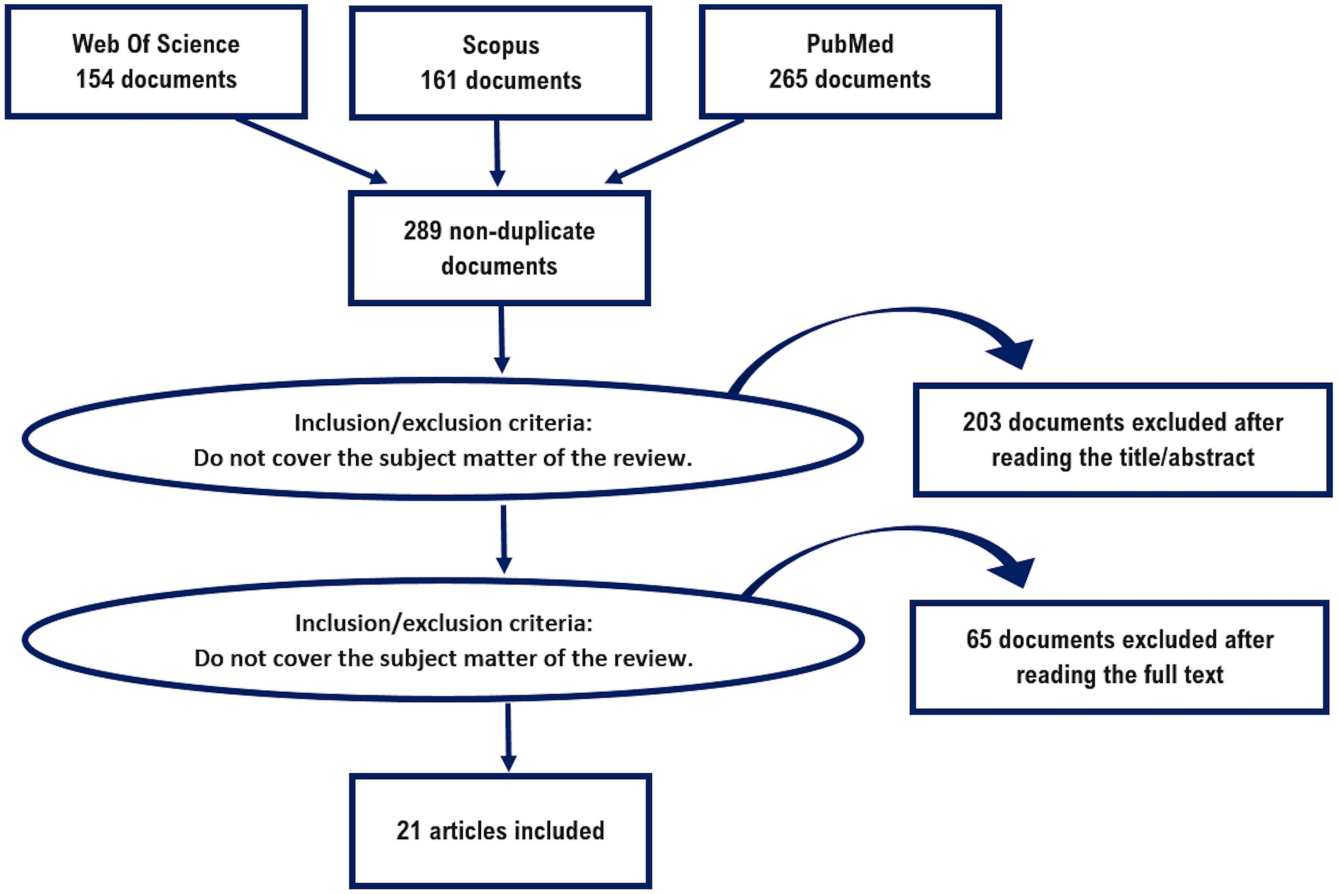
PRISMA diagram for this systematic review.

After deleting duplicate articles from the search process, a total of 289 potential articles were collected for inclusion in the study. Of these, 203 were excluded after reviewing their titles and abstracts because they were not related to the objectives of the review. Subsequently, a more thorough manual screening was performed, resulting in the identification of 21 articles fully meeting the pre-defined eligibility criteria for the study. Figure 1 illustrates the process of searching and selecting data sources.

### 3.2 Characteristics of eligible research articles

#### 3.2.1 Geographical coverage

The articles chosen span a publication period extending from 2015 to 2023, with the majority of them (*n* = 16; 76.2%) published in the last 3 years (i.e., 2020–2023), in accordance to an increased development of the market of ADAS during the last few years. Remarkably, all (100%) of the studies meeting the inclusion criteria (and consequently selected for analysis) were written in English.

**Figure 2:**
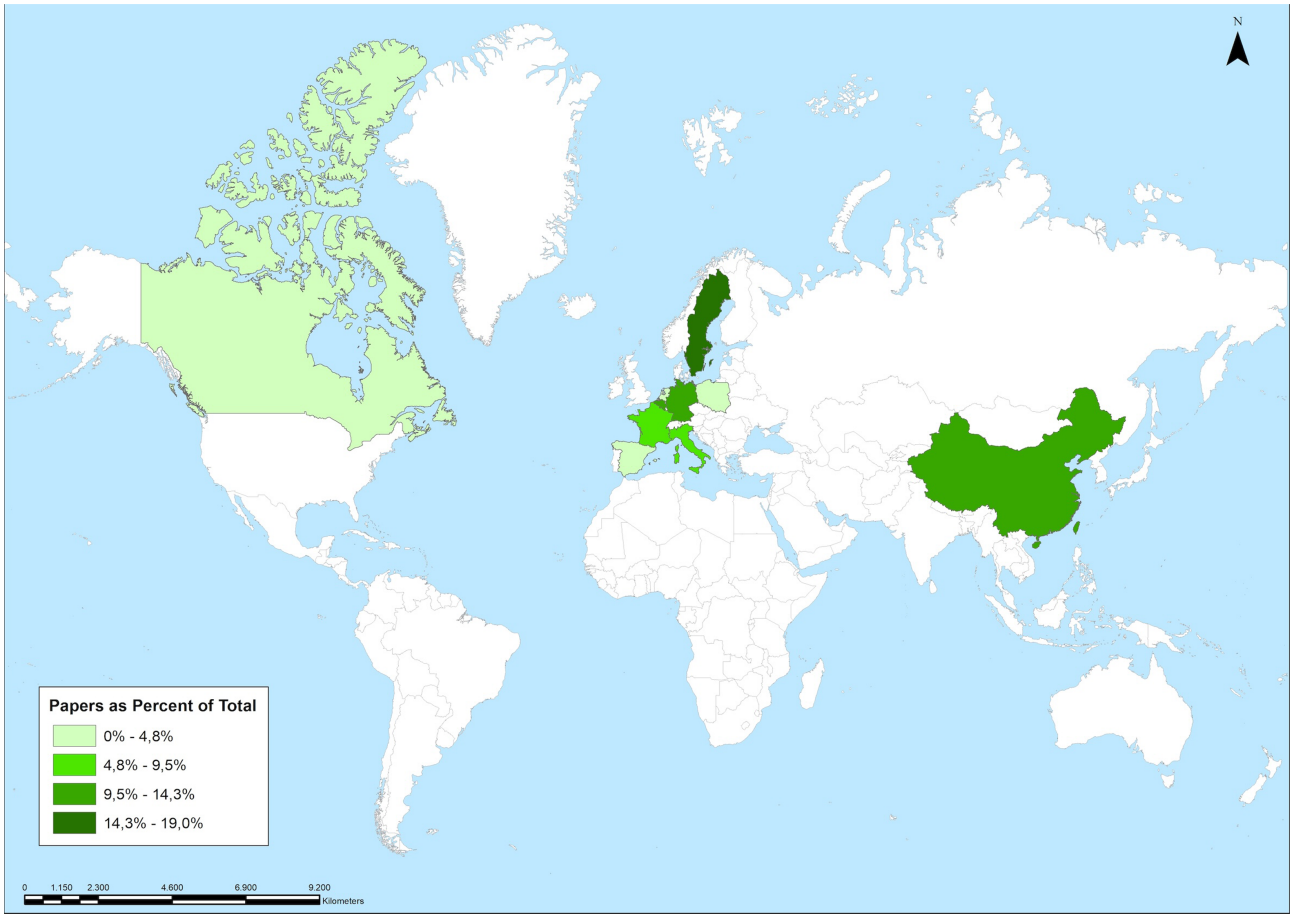
Geographical distribution (country of origin) of the selected studies.

In addition (in accordance with the aforementioned in section 2.3), the studies were conducted in different countries, as shown in Figure 2. After a basic frequency analysis, data sources represent a total of 10 countries located in three different continents, with the majority being from European countries (a total of 17 articles, equivalent to 80.9% of the total). Specifically, the distribution of countries is as follows: Sweden (*n* = 4), China (*n* = 3), Germany (*n* = 3), Belgium (*n* = 3), Italy (*n* = 2), France (*n* = 2), Spain (*n* = 1), Canada (*n* = 1), Poland (*n* = 1), and the Netherlands (*n* = 1).

#### 3.2.2 Subject matters

**Figure 3:**
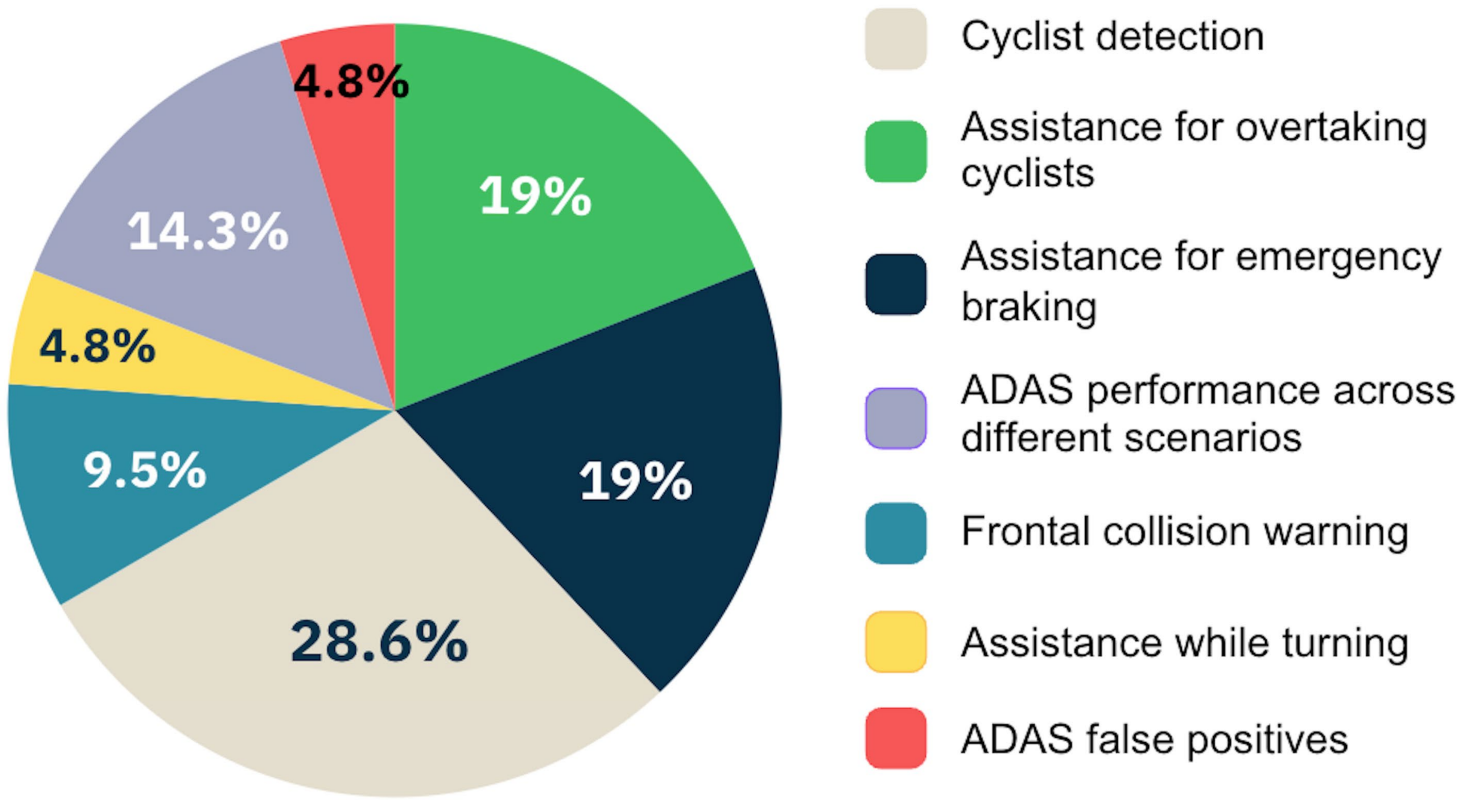
Groups of studies addressing different topics related to ADAS for preventing car-cyclist collisions, categorized by their subject matter (*N* = 21). The topics are listed in descending order of frequency, from the most common (cyclist detection) to less frequent themes (turning assistance and false positives).

In relation to the content and subject matter of the analyzed research outcomes, seven different groups were identified. Overall, most articles focus on evaluating specific ADAS devices performing analyses to determine their effectiveness or potential improvements after modification of certain parameters, even though with some specificities. The first (*n* = 4) delves into driver assistance technologies for overtaking cyclists, constituting 19% of the total. Similarly, a second set of studies (*n* = 4), making up 19% of the total, concentrates on vehicle emergency braking assistance devices. The third group (*n* = 6) focuses on evaluating the effectiveness of ADAS systems for detecting cyclists on the road, emerging as the most addressed topic among the appended ones, covering 28.6%. The fourth group (*n* = 2) centers on papers examining frontal collision warning devices in interactions with bicycle users (9.5%). The fifth group (*n* = 1) features a single paper evaluating a system designed to assist in turns without incidents involving cyclists, comprising 4.8%. The sixth group (*n* = 3) comprises articles that do not specifically evaluate ADAS systems but rather analyze a range of them in different scenarios to identify their effectiveness and potential enhancements in the active safety of motor vehicles. This group represents 14.3% of the papers. Lastly, the seventh group (*n* = 1) consists of a single article evaluating the false positives of Advanced Driver Assistance Systems, a critical factor influencing drivers’ behavior and perception of such systems (4.8%). Figure 3 visually illustrates the distribution of these paper groups as for their coverage among the analyzed results.

#### 3.2.3 Methodological setting

From a method-based approach (i.e., the study design used), most of the analyzed studies follow an experimental research design (71.42%, *n* = 15). Typically, an experimental methodology involves the creation of a control group, which represents the conditions without the intervention of ADAS devices, and an experimental condition, which operates with the assistance of these devices. Moreover, in some cases, it is possible to perform combinatory studies where the same group is subjected to two different phases: one without the devices and one with them, allowing direct comparison of their impact on the same set of subjects over time. In any case, within this type of methodology, three core groups or types of experiments can be identified:

On the one hand, those that are carried out in a controlled path by the researchers. This approach, which represents 38.09% (*n* = 8) of the total number of articles, is characterized by the fact that the research is carried out in a real road environment, but under controlled and safe conditions. Vehicles equipped with ADAS devices are used, real driving data are recorded, and relevant data are collected, such as speed, following distance, braking capacity or other parameters of interest.

On the other hand, 28.57% of the total research (*n* = 6) is conducted through driving simulators. In this approach, research is conducted in a virtual environment that simulates real traffic scenarios. Participants interact with a driving simulator that incorporates ADAS devices and are presented with a variety of traffic situations and driving scenarios, recording data similar to those obtained in a real context. Finally, one article uses a combined methodology, having performed measurements in a controlled route and simulated scenarios (4.76%, *n* = 1).

A small part of the investigations employs epidemiological methods (14.26%, *n* = 3). These are retrospective studies that use databases of road accidents from official entities or institutions and official figures on the presence, or lack thereof, of ADAS devices to evaluate the relationship between the two variables. Finally, some studies employ observational methods based on the visualization, recording and analysis of information in the real environment without the active intervention of the researchers. Thus, this type of research represents 14.26% (*n* = 3) of the total number of articles, and mostly corresponds to the observation and collection of data on a group of drivers using vehicles equipped with ADAS devices in everyday driving situations.

Table 2 shows the general characteristics of the analyzed original research articles.

**Table 2:** Record of the general characteristics of the selected studies.

Author (s), year of publication, country	Objectives	Methods and sample	Results (main outcomes)	Key limitations
Brijs et al., 2022 (Belgium) (47)	Design and evaluation of a driver assistance system for overtaking cyclists.	A sample of 48 drivers performed the established route with the system activated and deactivated.	The system had an impact on the duration of the overtaking phase, the lateral clearance in the overtaking phase and the hazard time in the process of the overtaking maneuver.	Repeated use of the system may reduce its effectiveness or cause learning effects that influence the results.
Siebke et al., 2023 (Germany) (48)	Emergency braking system (AEB) for cyclist detection in urban intersection scenarios is evaluated.	Driving simulations are used to evaluate 240,000 road situations with various sensor opening angles.	The presented approach allows examining the entire scenario space randomly, which minimizes the potential loss of information in risky situations.	No limitations are apparent
Rasch and Dozza, 2020 (Sweden) (49)	Design of a control model based on logistic regression to avoid false-positive ADAS activations.	Data from an experiment on a test track are recorded to establish the model.	It manifests limited ability to predict the probability and confidence of drivers braking and turning while approaching a cyclist during an overtaking, thus improving ADAS.	Small sample size
Kovaceva et al., 2022 (Sweden) (50)	The potential impact of forward collision warning systems on cyclist protection in overtaking situations is evaluated.	Drivers’ reactions to the warning were analyzed, combining the data with accident frequency and an injury risk model.	With the driver response model applied to the warning system, cyclist fatalities were reduced by 53 to 96% and serious injuries by 43 to 94%.	Simulations did not include responses other than braking (e.g., turning).
Schindler and Piccinini, 2021 (Sweden) (51)	The response of drivers in two conflict scenarios with vulnerable road users is analyzed.	A group of 13 people took a tour driving a truck equipped for data recording.	Drivers adapted their kinematic and visual behavior in situations where vulnerable road users were crossing the intersection, compared to the baseline route.	Small sample size
Char and Serre, 2020 (France) (52)	Analyzed accidents between cars and cyclists to determine potential improvements for vehicle active safety systems.	Analysis of 2,261 accidents involving cars and cyclists. Safety systems are applied in the most likely incident scenarios.	A field of view of 60° and a range of 35 m would allow detection of most cyclists in accident scenarios. With a 60° field of view, 51% of cyclists could be detected up to 4 s before the crash and 72% up to 1 s before.	The sample is not representative of the national proportion of accidents.
Limani et al., 2022 (Belgium) (53)	PowerCam, a system that enables compatibility between 802.11p and conventional Wi-Fi networks, is presented to connect cars with the cell phones of vulnerable users.	A standard wifi AP is included in the roadside unit’s broadcast radio and another just outside it to verify that messages are broadcast.	This methodology enables low bit-rate communication between devices without requiring formal association or authentication. The results demonstrate the system’s ability to deliver messages in a timely manner to users.	May have a latency of 2 s, not being effective in specific scenarios.
Cara et al., 2015 (Netherlands) (54)	A scenario classification algorithm using machine learning is proposed to evaluate ADAS systems for cyclist protection.	A data set consisting of 99 realistic cycling scenarios recorded by a vehicle equipped with instrumentation is obtained.	An accuracy of 87.9% is achieved in the classification of the data obtained, and the execution time of 45.8 microseconds supports the suitability of the algorithm for fine online applications.	No limitations are apparent
Puller et al., 2023 (Germany) (55)	A V2X-based turning aid designed to mitigate collisions with vulnerable participants in traffic is presented.	Generate information for advanced driver assistance systems to use, even when the sensors do not detect the object in the foreground, and increase awareness of crossings.	The application faces challenges in terms of user acceptance, so a key challenge for ADAS functions is to maintain a low false positive rate so that users do not lose confidence in its accuracy.	No limitations are apparent
Brijs et al., 2021 (Belgium) (56)	The impact of an advanced driver assistance system for cyclist overtaking is analyzed.	A driving simulator is used for the experiment in which there are three phases of warning priority: normal accident, hazard, and avoidable	A positive effect on lateral clearance was observed with ADAS presence, familiarity with the system, driving experience, and experience as a cyclist. A negative effect of cyclist maneuvering from the edge of the lane to the center of the lane, cyclists riding parallel, driver age, and self-reported aggressive driving.	No apparent limitations
Kovaceva et al., 2019 (Sweden) (57)	The combination of factors affecting the limits of drivers’ comfort zone when overtaking cyclists in a naturalistic environment is analyzed.	Naturalistic driving data from UDRIVE, a European naturalistic driving study, is analyzed.	The higher the speed of the car, the higher the driver’s comfort zone limits when approaching and passing, but the presence of an approaching vehicle decreases it when overtaking.	Limited generalization of the data set
Kovaceva et al., 2020 (Germany) (58)	The safety benefit of autonomous steering and emergency braking systems for the protection of cyclists and pedestrians is evaluated	Data from a simulation based on data from the German In-Depth Accident Study (GIDAS-PCM) were combined with real-world test results.	A systematic way of combining results from different sources is indicated, showing the positive effects of the evaluated system.	Other scenarios may require the application of an extension of the current model.
Anaya et al., 2015 (Spain) (59)	The effectiveness of V2X systems in detecting vulnerable road users is evaluated.	Two tests are performed to test the correct detection by the vehicle of both motorists and cyclists.	In both tests the vehicle correctly detects the vulnerable user even in blind spots when the distance between the two vehicles is less than 30 meters.	No limitations are evident
Guerrieri and Parla, 2021 (Italy) (60)	The aim is to obtain a program capable of detecting vulnerable road users by calculating their distance and speed, in order to be able to act from the streetcar.	Images obtained along the route of a streetcar are analyzed and processed by neural networks to obtain different parameters.	The system is able to correctly estimate the approach speed of pedestrians, cyclists and other vehicles.	No limitations are evident
Azadani and Boukerche, 2021 (Canada) (61)	The aim is to obtain the position of cyclists in motion in order to improve the detection capability of the ADAS.	Two different real scenarios are simulated in which the cyclist is in a position not visible from the vehicle, calculating his position.	In both scenarios, the ultrasonic sensors installed in different cars were able to locate the cyclist’s position and share the information among several vehicles to keep the cyclist located.	No limitations shown
Chen et al., 2018 (China) (62)	The improvement of pedestrian and cyclist identification by unifying 3 different detection methodologies is evaluated.	Evaluations of the proposed detection methodology are carried out by comparing it with other detection methods.	The proposed method shows a higher efficiency in recognizing pedestrians and cyclists than other methods used.	No apparent limitations
Ucińska and Pełka, 2021 (Poland) (63)	To analyze the effectiveness of the automatic braking system (AEB) in different situations in front of VRU.	44 trials of 4 tests are performed in which different scenarios are presented analyzing the AEB activity.	The different tests show the difficulty of the AEB system both in braking in time and in recognizing properly the VRUs present on the road.	Low sample size
Xu et al., 2020 (China) (64)	A neural network is trained to improve the differentiation between cyclists and motorists, as well as co-detection.	Comparison of the results obtained between the proposed method and the previously existing ones on several databases on the Internet consisting of more than one million images (4,300 of cyclists and motorists).	The results show a higher accuracy in differentiating between cyclists and motorists by up to 30%.	No apparent limitations
Duan et al., 2017 (China) (65)	Analyzing the braking behavior of drivers to improve the performance of ADAS braking systems on bicycles.	3 types of scenarios were simulated from the accident data collected and 25 drivers were tested.	The application of the data obtained can help to improve bicycle AEB systems and thus reduce the number of accidents.	Small sample size
Char et al., 2022 (France) (66)	Potential effects of earlier brake activation by drivers with a collision warning system are quantified in simulated car-to-cyclist accident scenarios.	A parametric analysis is performed by varying the field of view of the detection sensor, the activation time of the forward collision warning system and the reaction delay time of the driver to the forward collision warning system.	A 70° field of view, a system activation time of 2.6 s before impact and a driver reaction of 0.6 s to the warning system has a positive outcome in 82% of accident cases, with 78% avoiding and 4% mitigating the crash.	No apparent limitations

### 3.3 Content analysis: word communities

Regarding the discursive outlines of the analyzed studies, the content analysis software VOSviewer (67) has been used to detect the most relevant groupings or sets of words within the textual content of the articles we have chosen. This tool, designed principally for bibliometric analysis practices, serves as an efficient method to summarize and offer comprehensive syntheses of literature outputs, thus making it easy go know the state-of-affairs on a certain topic on the basis of the published literature. Consequently, and added to the positive fact of enhancing objectivity in the analysis of literature (68) it has gained popularity and widespread adoption in recent years due to its ubiquity and utility in simplifying the retrieval and evaluation of extensive volumes of scientific data (69).

**Figure 4:**
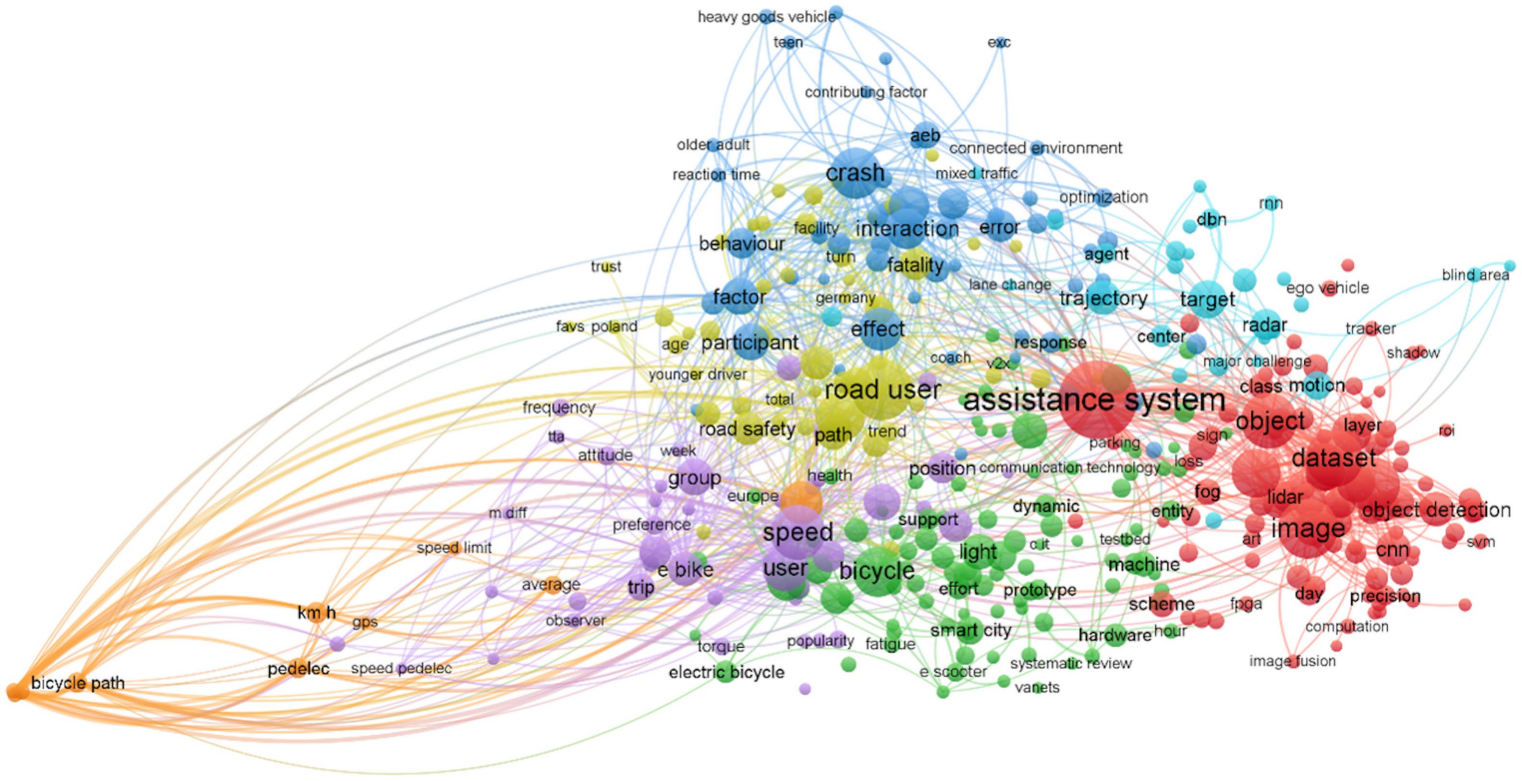
Representation of the clustered ‘word communities’ in the current empirical studies on the topic. Note: Each community is labeled with a different color, based on the number of commonalities and links identified in the analyzed literature sources.

Once the data was collected, the bibliometric information and the text corpus of all the selected documents was extracted in RIS format and applied in the VOSviewer software, which establishes common patterns, links, and builds word communities (AKA *word clusters*) on the basis of the full text contents. At a practical level, identifying these word clusters favors a succinct and easy recognition of central or main themes within a given set of documents. At a research one, this significantly aids in the efficient classification and organization of the selected collection of articles. Figure 4 graphically presents the groupings and connections between words in the texts, about which it is worth mentioning that all these selected terms appear in the individual text corpus of each source a minimum of five times.

After conducting the content analysis of the original scientific papers retained in this systematic review, several topics or ‘categories’ can be distinguished within the discourse they address. These specific topics have been grouped into seven core word communities, each with its own particular focus and meaning. Each word community is presented with a color code that facilitates its identification and understanding.

The word community in red relates to methodological aspects in the context of research on advanced driver assistance systems (ADAS) related to the interaction between cyclists and motor vehicles. It, therefore, refers to a specific study approach that focuses on the methods and techniques used to conduct research in this field. This grouping of words includes terms such as “assistance system,” “image,” “dataset,” and “object,” all of which are related to data collection, image analysis, dataset creation, and the evaluation of specific objects.

The community represented in blue focuses on factors related to crashes involving bicyclists and motor vehicles. Key terms in this category include “crash,” “interaction,” “effect,” “error,” and “contributing factor.” These terms reflect a discourse focused on road safety and, specifically, interactions between bicyclists and drivers, as well as the factors contributing to crashes and their consequences.

The green word community focuses on the devices and vehicles being studied in the research. Terms such as “bicycle,” “lights,” “electric bike,” “machine,” and “prototype” relate to the features and design of ADAS devices to investigate their impact on the safety of this vulnerable group.

The yellow word community is dedicated to exploring users and their characteristics. Terms such as “road user,” “road safety,” “younger driver,” and “age” indicate an interest in understanding the particularities of drivers who interact riskily with bicyclists, as well as those who use ADAS systems. Characteristics of bicycle users who experience incidents with motor vehicles are also apparent.

The purple community focuses on the perceptions, beliefs, and behaviors of users during their commute. Terms such as “speed,” “attitude,” “preference,” “trip,” “frequency,” and “group” highlight the importance of understanding how user attitudes and behaviors can affect the implementation and effectiveness of ADAS systems. This area of study focuses on decisional aspects of users interacting with these systems and how they influence the prevalence of their use and acceptance.

The community in orange differs from the rest, as it focuses on a specific element, the “bicycle path.” This separation is justified because most studies focused on interurban environments with no bicycle lanes, and cyclists share the road with motor vehicles. In this sense, this difference in discourse, as well as the small number of articles that address this topic, explain why graphically fewer relationships are identified between this word community and the rest of the groupings.

Taken together, these word communities represent the different areas of focus in research on ADAS systems and cyclist-motor vehicle interaction, providing a deeper understanding of the key issues and questions that researchers address in this field.

### 3.4 Evaluation of the quality of the selected studies

**Figure 5:**
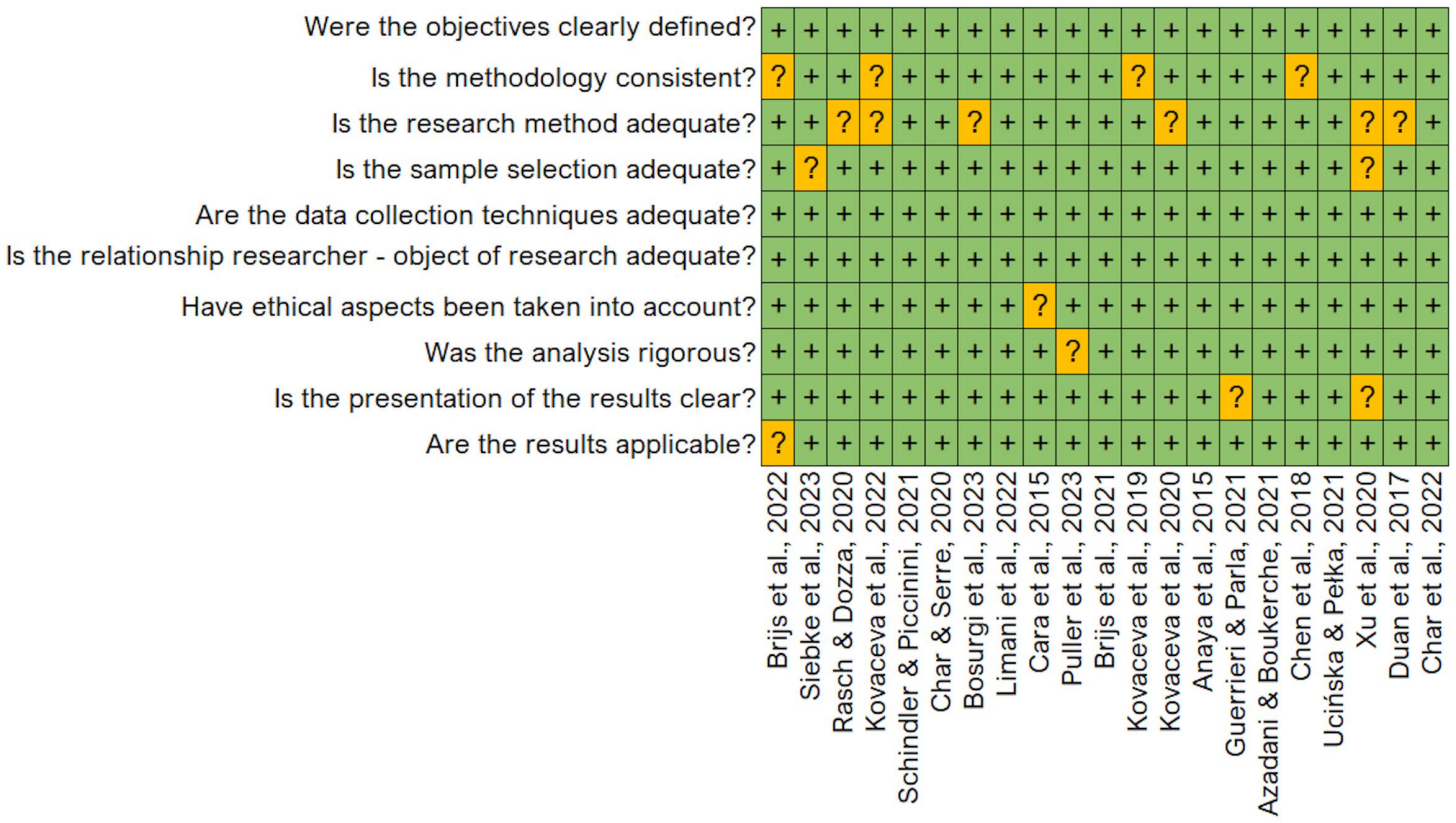
Evaluation of the quality of the selected articles using the “Critical Appraisal Skills Programme” tool.

The quality assessment methodology provided by the Critical Appraisal Skills Program (CASP) was used to ensure that no included study had the capacity to influence or distort the results of this systematic review. Through a set of 10 specific questions, this tool allows the evaluators to assess the level of rigor, reliability and relevance of each study (70). The CASP focuses on the development of practical skills for critical evaluation, being an instrument easily adaptable to different types of studies and simple to apply, being valid for evaluating qualitative, quantitative and mixed studies. The results of the evaluation of the selected publications are shown in Figure 5. It is important to mention that all studies were included in the review because of their low risk of bias, and no articles previously selected in the screening phase were excluded in this process.

## 4 Discussion

In the current context of road safety, the interaction between motor vehicles and cyclists has become an issue of significant concern. Advanced driver assistance systems (ADAS) have the potential to be effective tools for reducing collisions between drivers and cyclists. For these reasons, the aim of the present systematic review has been to target and analyze the existing research analyzing the effectiveness of this type of devices, specifically for the prevention of crashes involving cyclists, and to synthesize their main results.

In terms of quantity (i.e., current volume of scientific production), it should be noted that there is still a small number of studies that analyze this specific topic, even though their overall quality has been found reasonably well after applying the selected evaluation methods. Also, it is worth mentioning that, although there is a relatively large number of articles evaluating ADAS devices on vulnerable groups, these are more focused on pedestrians or motorcyclists (71, 72). One of the inclusion criteria of the present review is that the selected research should specifically include results on cyclists, which reduces the number of potentially selectable articles, but helps to match the findings to the characteristics of this road group.

### 4.1 Effectiveness of ADAS systems in preventing collisions with cyclists

As evidenced, ADAS systems encompass a wide taxonomical variety of technologies, typically ranging from forward collision alerts and automatic emergency braking systems to blind spot detection systems and lane departure prevention assistance. The majority of selected researches point out that the integration of these functions in motor vehicles allows preventing collisions with cyclists in a broad number of situations (49, 52, 56, 58). The results of the different studies manifest that the models and devices evaluated, allow increases in terms of drivers’ risk recognition, detection of cyclists, and visual field. On the other hand, these ADAS have been shown to imply a reduction in mean reaction times, thus contributing to reducing both fatalities and serious injuries derived from collisions involving bicycle riders (59, 62, 66).

However, conflicting factors have been also underscored through different pieces of literature, making it relevant to mention that there are variables that may influence the effectiveness of ADAS systems related to driver characteristics and the road situation or environment. For instance, Brijs et al. (47) note that driver age and self-reported aggressive driving were variables that negatively influenced overtaking performance, despite having an in-vehicle collision warning system. A possible explanation lies in the tendency of drivers with these characteristics to ignore or minimize the alerts and warnings provided by the system (73). Also, drivers who self-identify as aggressive have been found to tend to be more prone to take risks and underestimate the importance of warning signals (74). This may lead them to underestimate the proximity of cyclists and make risky decisions enhanced by psychosocial factors such as fatigue and stress, performing less safer overtaking maneuvers and thus increasing the risk of collision (75, 76).

In contrast, the variables of experience as a car driver and experience as a bicycle user had a positive impact on driver behavior during overtaking. Specifically, when these parameters were high, in the presence of the ADAS system, the lateral separation distance between the vehicle and the cyclist maintained by the driver significantly increased (47). In this regard, especially experience as a cyclist provides a unique perspective in understanding the vulnerabilities of those who commute by bicycle (77). Drivers with experience as cyclists are often more cautious when overtaking such users, as they understand firsthand safety concerns, such as sufficient space to maneuver or the possibility of being affected by wind gusts created by vehicles passing at high speed (78).

A positive effect of familiarity with the ADAS system on driver behavior has also been evidenced. This variable contributes to a more intuitive interaction with the system, and, consequently, decreases the probability of making errors or infractions in vehicle operation (79). This finding is congruent with previous literature. The predisposition to the use of technological devices while driving has been related to the perception of technology in general at both the individual and collective levels (80). Thus, if a given region or cultural context presents a high familiarity and experience with ICTs, they are more likely to rely on these devices in a traffic context (81, 82). On the contrary, in emerging countries where there is a lower presence of technological tools, there is a greater rejection of the use of ADAS or ITS systems while driving, which may be due to elements such as the perceived lack of privacy of personal data or the lack of knowledge about their functionalities (83).

In relation to the road environment, some of the articles included in this review also point out that the complexity of the scenario plays a fundamental role in the degree of usefulness and effectiveness of ADAS systems. Bosurgi et al. (79) point out that it is difficult to determine quantitatively and in general terms the effectiveness of these driving aids. In this sense, they point out that in many road situations, the information transmitted to the driver by an onboard ADAS system enhances the maintenance of adequate behavior and an improvement in driving performance. However, in complex scenarios such as when there are special traffic components or difficult weather conditions, drivers pay less attention to the information and alerts from such systems, which is estimated to occur because the user reaches the limit of his or her capacity to process additional information (55, 65).

### 4.2 Impact of ADAS ‘false alarms’ and other relevant issues related to driver behavior

One *side-effect* commonly addressed in the literature on ADAS systems is how drivers may become over-reliant on technology and develop the expectation that they will always detect and avoid hazards (84, 85). This could lead to distraction and negligence, as drivers may take their attention away from the road and become more easily distracted by electronic devices, conversations, or other non-driving activities, assuming that the technology will take care of everything (86–88). This factor can be dangerous and contradicts the fundamental purpose of ADAS, which is to assist drivers, not replace their responsibility for safe driving (89).

Other studies focused on relevant safety-related matters such as drivers’ aptitudes have also underscored the potential (and negative) effect of technology misuse and misunderstanding, especially among drivers with poor aptitudes or engaging in problematic driving behavior besides the safety potential of Advanced Driving Assistance Systems (90). Moreover, other previous studies have explored the association between drivers’ cognitive abilities and decision-making skills in visually impaired drivers, finding that not all of them have the same usefulness and acceptance level (91). Additionally, prospective evidence show the need to include ADAS in drivers’ training curricula, and not fully depending of technology as a ‘driver behavioral management’ tool, as problematic driving behaviors, such as aggressive driving or distracted driving, can create additional challenges for ADAS in predicting and mitigating potential road risks (92, 93).

In this sense, ADAS systems have been demonstrated to be, besides relatively reliable, never infallible. Their accuracy and reliability may vary depending on factors such as weather conditions and sensor quality (94). A crucial aspect of the effectiveness of these devices lies in the sensitivity and accuracy of their alerts. Thus, false alarms generated by advanced driver assistance systems pose significant challenges in the context of cyclist crash prevention (95). When these systems issue incorrect hazard alarms, inappropriate driver responses can be triggered. These responses may include abrupt maneuvers, sudden braking, or unnecessary avoidance, which in turn may increase the risk of collisions, particularly rear-end collisions, or hazardous situations with other vehicles on the road (96).

On the other hand, another potential consequence of false alarms in ADAS systems, noted in several selected articles in the present review, is driver desensitization (50, 51). When these devices have these errors on a regular basis, drivers may become less responsive to alerts. They become habituated to the situation and may begin to ignore them or fail to respond effectively, even when a legitimate alarm is triggered. This reduces the usefulness of system alerts and can increase the risk of not reacting appropriately in real danger situations. In addition, the false accuracy of warnings directly influences the perception of users, their acceptance of these tools and, their predisposition to want to implement ADAS systems in their vehicles (52). Therefore, these devices must be able to reliably distinguish between a real cyclist and other objects or situations on the road in order to avoid unnecessary alarms that may desensitize drivers (97).

### 4.3 Complementary measures to reduce vehicle-cyclist incidents

In addition to ADAS systems that can be implemented in motor vehicles, it is essential to consider other both technological and non-technological measures that can have a significant impact on road safety and accident reduction between cars and cyclists. To the best of our knowledge, there is no research on the effectiveness of cyclist assistance systems. It is less common to find ADAS systems specifically designed for bicycles, as bicycles are often not equipped with the same technology as motor vehicles. Instead of ADAS systems, cyclists often use other devices and applications designed to enhance their safety and visibility on the road such as lights and reflectors, action cameras, rearview mirrors, and navigation and communication devices that allow them to alert their position to other road users (98). However, given the potential of ADAS systems, more effort could be devoted to designing devices that could be used by cyclists themselves to alert them to road hazards in real-time, providing them with information that would allow them to adapt as far as possible, their behavior to the road scenario.

Another promising approach is the implementation of vehicle-to-vehicle (V2V) and vehicle-to-infrastructure (V2I) sensor and communication systems (99). These advanced technologies focus on creating a real-time information network involving all traffic components, which could revolutionize road safety for the benefit of all road users, including cyclists. V2V communication allows vehicles to exchange critical data, such as position, speed, direction, and operational status (100). When applied to situations with cyclists, these systems can detect cyclists in close proximity and provide warnings to drivers. On the other hand, V2I communication involves the interaction of vehicles with road infrastructure, such as traffic lights and traffic signals (101). This could enable the synchronization of traffic lights to ensure safe passage for cyclists or the detection of cyclists at dangerous intersections, which could result in automatically triggered warning signals to protect cyclists.

Complementary to the application of technology, other sources claim (from the non-technological point of view) the need for promoting both drivers’ training on potential road conflicts and awareness about the importance of sharing the road safely with cyclists (102). The implementation of driver assistance devices is a significant step, but responsibility and mutual respect on the road are equally essential to ensure the safety of all users. In fact, Wood et al. (103) point out the importance of training for both drivers and cyclists, as it is indicated that the two user groups have divergent perceptions of responsibility for car-cyclist collisions. Therefore, road safety education programs and communication campaigns on cyclist safety, supported by real-time information systems and applications, can play a key role in reducing accidents. In fact, research suggests that different preventive measures significantly increase their effectiveness when implemented in a coordinated and complementary manner (104).

In summary, although our study reveals a scarcity of developed evidence on the effectiveness of Advanced Driver Assistance Systems (ADAS) for preventing car-cyclist crashes, particularly in a limited number of countries, especially those with high income, literature uniformly emphasizes that the rise in the number of crashes involving cyclists is a global problem affecting all regions of the world. The implementation of driver assistance devices that alert to the presence of cyclists or act upon interaction with these users is presented as an effective solution to improve road safety in many driving scenarios. When implemented effectively, this technology can reduce conflicts and risks associated with overtaking cyclists and save lives globally. However, as previously mentioned, driver perception and experience influence their willingness to use ADAS systems, as well as their behavior once alerted. Therefore, it is equally important to encourage education and respect between drivers and cyclists in order to create a safe and harmonious driving environment for everyone.

### 4.4 Limitations of the systematic review

This systematic review was performed following the PRISMA procedure to avoid possible biases in the selection and/or recording of data. In addition, the eligible articles are part of relevant indexes and databases recognized by the scientific community and the CASP instrument has been applied to guarantee, as far as possible, the quality of the research. Despite their methodological rigor, all systematic reviews have some inherent limitations that should be taken into account.

On the one hand, publication bias may occur, especially highlighting the fact that studies with positive results tend to be more likely to be published than those reporting inconclusive results (105). Therefore, there may be studies with negative or non-significant results that, since they are not published, cannot be included in the review performed. Moreover, previous studies have remarked on the relevance of addressing selection bias (e.g., gender and outcome-related bias) as a confounding factor, which consists of the selective inclusion of studies based on certain criteria stipulated by those responsible for searching and selecting the articles (105, 106). This bias has been minimized by all the authors of the article carrying out this process individually and independently. Additionally, bias occurs in matters of language and region of publication (107). The present systematic review focuses on research published in English and Spanish, so potential studies relevant to the research topic that have been published in other languages have not been included. Further, it is noteworthy that key issues such as ADAS-related behavioral ergonomics or their usage patterns over time (e.g., from a longitudinal point of view) remain understudied, implying an active need for further research and developments in this field.

Finally, it is worth addressing the fact that, in the discourse analysis conducted with the VOSviewer tool, the absence of combining synonyms and the use of different notations for some terms imposes limitations on lexical richness. For example, and among many others, electric bikes may be labeled across literature sources as e-bike, e-bicycle, electric bicycle, electronic bicycle, and so on. These acute but relevant differences may compromise the full word clustering process and its holistic interpretation, leading to a loss of linguistic subtleties. In addition, specialized scientometric sources (68) emphasize that the precision of bibliometric analyses is significantly contingent on both the breadth and quality of data. Given the current reliance on a limited number of available sources, this can influence the overall accuracy. Furthermore, interdisciplinary research, as observed in the present study, often involves different paradigms and approaches, introducing additional challenges to the precision of scientometric and bibliometric analyses. This must be considered when interpreting the outcomes of the present research.

## 5 Conclusion

This systematic literature review aimed to analyze the evidence regarding the effectiveness of in-vehicle Advanced Driver Assistance Systems (ADAS) in preventing car-cyclist collisions. The results of this review lead to the following conclusions:

First, in terms of the volume of scientific production, there are relatively few studies that specifically address this topic with cyclists. Most studies tend to focus on pedestrians or motorcyclists. However, the overall quality of the available studies has been reasonably good, as determined by the selected evaluation methods.

Secondly, the literature supports the value of in-vehicle ADAS for preventing car-cyclist crashes. Nonetheless, these studies also highlight potential side effects, including unrealistic expectations of these systems and drivers’ overconfidence or desensitization, which should be regarded as latent threats.

Thirdly, regarding complementary non-technological factors that could enhance the effectiveness of ADAS in preventing car-cyclist crashes, there is a need to increase driver awareness and address potential inter-user conflicts through educational and training initiatives.

Finally, future research should encompass emerging issues such as behavioral ergonomics and the long-term effectiveness assessments of ADAS in preventing car-cycling crashes and their subsequent injuries.

## Data availability statement

The original contributions presented in the study are included in the article/supplementary material, further inquiries can be directed to the corresponding author.

## Author contributions

SU: Conceptualization, Data curation, Formal analysis, Investigation, Methodology, Project administration, Supervision, Validation, Visualization, Writing – original draft, Writing – review & editing. MF: Data curation, Formal analysis, Investigation, Methodology, Visualization, Writing – original draft, Writing – review & editing. FA: Conceptualization, Funding acquisition, Investigation, Methodology, Resources, Software, Supervision, Validation, Visualization, Writing – original draft.
